# Integrating behavioral science into urban planning: a framework for human-centered spatial design

**DOI:** 10.3389/fpsyg.2025.1632523

**Published:** 2025-08-12

**Authors:** Mohamed M. E. Khogali, Eman Ahmed Mohamed Ali, Abbas Ramdani

**Affiliations:** ^1^Department of Town Planning & Survey, Sharjah, United Arab Emirates; ^2^Department of Sociology, College of Art, Humanities and Social Sciences, University of Khorfakkan, Khorfakkan, Sharjah, United Arab Emirates; ^3^Department of Communication, College of Art, Humanities and Social Sciences, University of Khorfakkan, Khorfakkan, Sharjah, United Arab Emirates

**Keywords:** urban planning, behavioral science, environmental psychology, human-centered design, spatial behavior

## Abstract

This paper examines the intrinsic relationship between urban planning and behavioral concepts, addressing a significant knowledge gap in how spatial arrangements influence human behavior and well-being. Through a systematic literature review and analytical framework, we investigate the interdependence between urban spatial design and human behavioral patterns across seven key planning domains: urban form, built environment, infrastructure services, urban landscapes, public spaces, urban housing fabric, and urban design. Our findings reveal that physical environments significantly shape human psychological, cognitive, and behavioral responses, while human activities simultaneously influence urban structures. The research identifies critical behavioral determinants that impact urban environments and demonstrates how behavioral science can reduce uncertainty in design processes. By integrating insights from environmental psychology and behavioral theory, this study offers a conceptual model to guide urban planners toward more behaviorally responsive design approaches. We conclude that well-planned cities support economically prosperous, socially cohesive, and environmentally sustainable communities, while poorly planned environments can exacerbate social unrest and hinder development. The study recommends institutionalizing participatory design methodologies, prioritizing pedestrian-oriented layouts, equitably distributing green spaces, adopting context-sensitive planning approaches, and implementing rigorous post-implementation behavioral assessments to develop truly human-centered urban environments.

## Introduction

Integration of behavioral principles in urban planning has developed considerably since 2020: researchers have documented specific applications of behavioral insight into practice. Behavioral science should be systematically integrated with urban planning, as urban forms serve as architecturally important alternatives to make it easier for people to make climate-friendly choices and improve the vibrancy of the city (Nieuwenhuijsen et al., 2024). This represents a departure from the traditional rational option model that dominated the planning theory of the mid-20th century. Recent research has docked the average impact of behavioral interventions in urban contexts. Between major urbanization and technology processes, scholars are looking for innovative concepts and methods to study the urban environment (Shilon and Eizenberg, 2024).

These studies employ advanced behavior mapping techniques that track real user behavior in urban places, walking beyond survey-based approaches to catch real-time spatial decision-making patterns. The theoretical basis for practical urban planning is drawn from research established in environmental psychology and spatial cognition, but recent work has developed a more sophisticated framework. Interdisciplinary research is becoming increasingly important to deal with complex problems like climate change as a major factor in human behavior that needs to be considered to find solutions (Verplanken et al., 2022). This approach systematically addresses cognitive boundaries, social impacts, and emotional reactions that affect spatial decision-making processes.

Contemporary applications display quantitative relations between urban design intervention and behavioral results. Research has established reasons between street design modifications and pedestrian behavior changes, public space configuration and social interaction patterns, and transit system design elements and ridership decisions. The concept of “active problem solution” synthesizes the integration of many factors and may represent the complexity of the event (Viale, 2024), suggesting that behavioral reactions to the urban atmosphere include complex cognitive processes rather than simple stimulus–response mechanisms in the behavioral environment.

The area now assumes that urban form acts as an architecture of choice that affects daily behavioral decisions. Studies have determined how embedded environmental signals, spatial obstacles, and social signaling system location options are in mobility patterns and social behavior in urban design. Although the systematic integration of these behavioral insights in comprehensive planning exercises remains limited, special applications are concentrated in special domains such as transport schemes and public space design.

### Problem statement

Urban environments are not neutral spaces: they are active agents in shaping and influencing human behavior. Despite the increasing integration of psychological and sociological insights into urban planning theory, a disconnect still exists between how cities are designed and how individuals interact within those spaces. Most traditional urban planning models prioritize functionality, infrastructure, and aesthetics without adequately accounting for the lived experiences, emotional responses, and behavioral patterns of urban dwellers.

There is a notable knowledge gap in understanding how spatial arrangements, architectural forms, and planning policies either support or hinder human well-being, social interaction, and behavioral development. While research in environmental psychology and behavioral geography has made progress, urban planners often lack practical frameworks to translate this knowledge into tangible design strategies.

This study addresses the critical need to explore and articulate the complementary relationship between urban planning and human behavior. By bridging the divide between spatial design and behavioral science, the research aims to contribute to the development of cities that are not only efficient and sustainable but also psychologically and socially responsive.

### Research objectives

We aim to examine the interdependent relationship between urban planning practices and human behavior, and to identify how spatial design can positively influence social and psychological well-being in urban environments.

The research objectives are as follows:

1. To analyze the impact of urban spatial configurations on individual and collective behavior
2. To explore the psychological and social responses elicited by various urban forms and environments
3. To evaluate current urban planning models in terms of their responsiveness to human behavioral needs
4. To identify design principles that enhance human interaction, comfort, and mental health in cities
5. To offer recommendations for integrating behavioral insights into urban planning processes

### Research questions

*Main research question*:

- How does urban planning influence human behavior, and in what ways can spatial design be optimized to foster positive psychological and social outcomes?

*Sub-questions*

1. What behavioral patterns are associated with different types of urban environments (e.g., high-density vs. low-density areas)?
2. How do elements such as public space, walkability, and green infrastructure affect social interaction and emotional well-being?
3. To what extent do existing planning policies incorporate behavioral research findings?
4. How can urban planning be improved to support inclusive, safe, and behaviorally supportive communities?

### Significance of the study

#### Academic significance

This research contributes to the growing interdisciplinary dialogue between urban planning and behavioral sciences. It offers a theoretical framework for understanding the reciprocal dynamics between space and behavior, thereby enriching urban studies with psychological and sociological dimensions.

#### Practical significance

For urban planners, architects, municipal authorities, and policymakers, the study provides actionable insights for designing cities that better accommodate human behavioral needs. The findings can inform zoning laws, design standards, and community development strategies that are more humane and socially adaptive.

#### Societal or community-level benefits

Urban environments that are behaviorally attuned can enhance quality of life, foster community cohesion, reduce stress and social isolation, and promote healthier lifestyles. This study serves as a step toward developing inclusive, resilient, and responsive cities that truly serve their inhabitants.

To ensure conceptual clarity and strengthen the interdisciplinary basis of this research, several key theoretical constructs are briefly defined below. These definitions serve as the analytical lens through which behavioral influences on urban design are explored.

- The *complementary relationship* between urban planning and behavioral science refers to a reciprocal interaction in which spatial configurations influence human action, and behavioral insights improve spatial planning outcomes. This integration highlights the need for planning strategies that account not only for structural and aesthetic factors but also for cognitive, emotional, and social dimensions of human experience (Donaghy and Hopkins, 2006a,b).
- *Abnormal behavior*, as discussed in environmental psychology and urban studies, refers to patterns of behavior that deviate significantly from social norms in a given space. These may manifest as avoidance, aggression, or distress in response to environmental stressors, often revealing design failures or psychological mismatches within urban settings (Lang, 1987).
- *Self-Determination Theory* (SDT), developed by Deci and Ryan (1985), posits that human motivation is driven by the fulfillment of three innate psychological needs: autonomy, competence, and relatedness. In urban contexts, SDT helps explain how environments that support these needs foster greater engagement, belonging, and well-being.
- *Prospect Theory*, introduced by Kahneman and Tversky (1979), challenges classical utility theory by showing that individuals tend to evaluate outcomes relative to a reference point and disproportionately avoid losses over acquiring gains. This theory provides a behavioral basis for understanding risk perception and decision-making in urban movement and spatial use.
- *Bayesian Theory*, in the context of spatial cognition and behavioral modeling, refers to the process by which individuals update beliefs and expectations based on new spatial information. Bayesian reasoning underpins how humans infer environmental affordances and adjust behavior in uncertain or novel urban environments (Tenenbaum et al., 2011).
- Finally, Biophilia refers to the innate human affinity for nature and living systems, a concept that increasingly informs urban design strategies focused on psychological restoration, resilience, and sustainability (Wilson, 1984; Kellert, 2008). Integrating biophilic principles into urban planning offers promising pathways to align spatial development with human emotional and ecological needs.

## Methodology

Sociology, psychology, and urban planning intersect in many important aspects, as these fields focus on understanding and improving environments and human interactions. The intersection of both sociology and behavioral sciences with urban planning is crucial to creating vibrant, safe, comfortable, equitable, and sustainable urban environments. By incorporating sociological and behavioral perspectives into planning processes, urban planners can better address the complex social issues that arise in urban environments, ultimately leading to more effective and inclusive outcomes. In this article, we set out to establish a deeper understanding of the relationship between urban planning and social and behavioral concepts in general by delving deeper into the published literature. To understand and embody this relationship more deeply, we examined the behavioral concepts and theories used in urban planning.

### Literature review methodology

This study employed a *systematic literature review* to examine the integration of behavioral science principles into urban planning. The methodology was designed to ensure transparency, rigor, and reproducibility in line with PRISMA (Preferred Reporting Items for Systematic Reviews and Meta-Analyses) guidelines (Page et al., 2021).

### Search strategy and databases

A comprehensive search was conducted across major academic databases, including Scopus, Web of Science, PubMed, PsycINFO, and Google Scholar. These databases were selected to cover a wide range of disciplines relevant to behavioral science and urban planning.

The search strategy combined keywords and Boolean operators tailored to capture studies at the intersection of behavioral theories and urban spatial design. Examples of key terms included the following:

- “behavioral science,” “environmental psychology,” and “behavioral interventions”
- “urban planning,” “urban design,” and “built environment”
- “human behavior,” “behavior change,” “place attachment,” “nudging,” and “walkability”

Only English-language, peer-reviewed publications from 2010 to 2023 were considered to ensure relevance and scholarly quality.

### Inclusion and exclusion criteria

#### Inclusion criteria

- Scholarly articles, book chapters, or conference papers
- Studies linking behavioral science theories or interventions with urban planning practices
- Empirical or conceptual research focusing on human behavior in urban contexts

#### Exclusion criteria

- Works limited to technical, economic, or engineering perspectives without behavioral insights
- Studies focused exclusively on non-urban or rural contexts
- Editorials, opinion pieces, or non-peer-reviewed materials
- Studies lacking clear relevance to behavior in spatial environments

### Screening and selection process

The screening process was conducted in multiple phases. After initial database searches, duplicates were removed, and the remaining records underwent title and abstract screening by two independent reviewers. Articles that passed this stage were then assessed at the full-text level using the predefined inclusion criteria.

Disagreements between reviewers were resolved through consensus discussions. Inter-rater reliability was calculated using a standardized statistical method, indicating strong consistency in the review process. The final group of studies selected through this rigorous evaluation served as the foundation for data extraction and thematic synthesis.

### Data extraction and thematic analysis

Key data were systematically extracted from each included study using a structured form. This captured study characteristics, theoretical frameworks, planning domains addressed, methods, and main findings.

An *inductive thematic analysis* approach (Braun and Clarke, 2006) was used to identify recurring behavioral themes and conceptual patterns across the literature. These themes were organized into major domains:

- Behavioral interventions in transportation and mobility
- Environmental psychology and spatial behavior
- Place identity and attachment in neighborhood design
- Nudging strategies in urban policy
- Community engagement and social cohesion in planning

**Figure 1:**
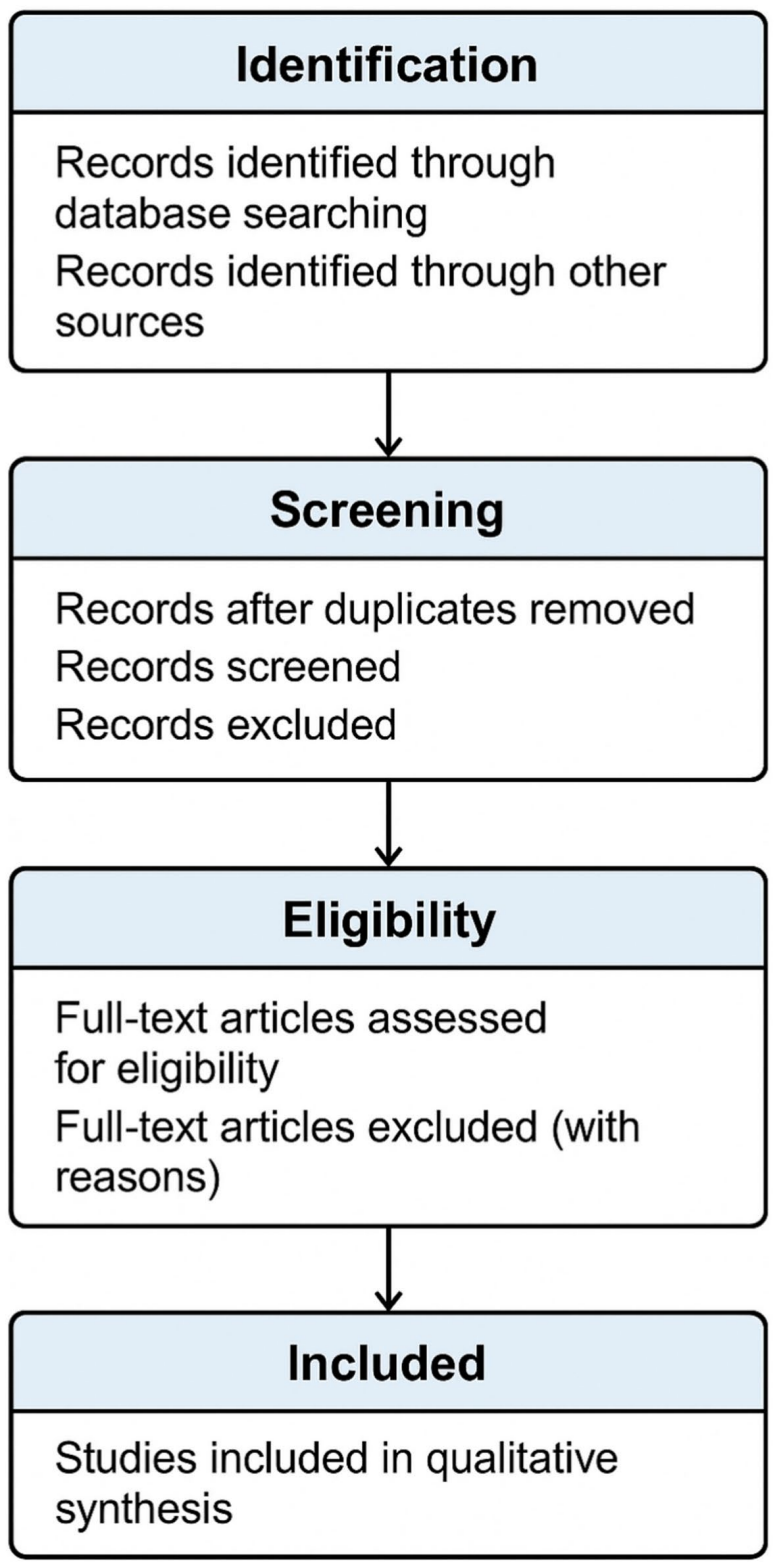
PRISMA flow diagram depicting the stages of the systematic literature review process.

This synthesis informed the conceptual framework presented in this study and underpinned the recommendations for behaviorally informed urban design (see Figure 1).

### Theoretical framework

Of interest to urban planners, abnormal behaviors are generally divided into individual behavior at a micro level to group behavior at a macro level. As noted by Donaghy and Hopkins (2006a,b), effective urban planning can be informed by good human behavior theories, as they guide spatial planning activities. Sociology and behavior theory can be applied to the behavioral levels of urban planning, for example, through theoretical applications: e.g., the rational actor model, the organizational process model, and the collective action theory of collective behavior by Olson (1971), Schulz and Stifel (2010), and Zellner and Campbell (2015). Behavioral intervention theory applications, such as nudge theory, generally occur at an individual change level, while applications such as change theory occur at a level higher than individuals.

Other studies, e.g., Alexander (1987), focus on place psychology, particularly the concept of “mental maps” carried by individuals as internal guides to urban space. Individuals gather sensory information to assess whether a place feels safe, comfort-providing, active, peaceful, or threatening. Numerous physical qualities, such as form, scale, landmarks, landscape, places, open space, landscape, and other spatial elements, contribute to a place’s overall character. When these elements are properly combined, as well as when factored in along with place psychology, they lead to urban quality. Urban quality, however, is not determined by physical factors alone; it also relies heavily on social, psychological, and cultural dimensions of place. In urban form, especially as it relates to human activity, the appeal of a place-oriented understanding and approach assumes that places serve as agents of learning, community-building, and social capital. Thus, the essence of place, how people observe and interact with space, and what incentives exist for placemaking are vital elements of urban design.

### Behavioral planning theories in urban planning models

A multidimensional set of behaviors relevant to urban planning requires investigation of the rational, cognitive, economic and psychological facets among others embedded in human mental frameworks. These facets of the same vary in their effects on the effectiveness of specific behaviors relative to the action in question. Collectively, however, these aspects aim to streamline efforts towards realizing the most optimal outcomes during the decision-making process. Compared to traditional methods used in urban modeling that mainly focus on quantitative methods, the use of spatio-temporal modeling frameworks in dynamic microsimulation, e.g., modeling based on an agent (ABM), automata of cell (CA), and neural connections (NC), provides powerful tools in implementing multi-method studies. This aims to reveal individual behaviors in the context of urban planning (Kwon and Silva, 2023):

- Fundamentally, dynamic simulation enables both creative induction and deduction. It produces simulated data that must be analyzed inductively—a process called generative social science (Epstein, 1999).
- Second, these tools provide a platform that facilitates quantitative and qualitative data, equation-based methods, and language-based methods (Yang and Gilbert, 2008), while computer packages enable more language-based processing.
- Third, the simulation dynamic tools are very powerful tools in the hands of spatial planners because they can interact with data from geographical systems (Brown et al., 2005), as has been done in a range of models.

However, by reviewing the literature, it could be argued that many planning disciplines are concerned with different attitudes, clarified through various types of theories. Although difficult to categorize as a behavioral theory, every discipline working in urban planning utilizes behavioral concepts alongside theory: for example, the probability theory.

Ideas, in turn, are important determinants of human behavior. Behavioral determinants consist of various social factors, such as trust, societal norms, general beliefs, group norms, social interaction, and general knowledge; this also includes individual characteristics, such as values, attitudes, fears, habits, practical problem-solving skills, motivation, ideas, and self-conceptualization. Moreover, statistical tests can be applied to examine and evaluate various aspects of urban planning.

The following table outlines the major social and behavioral theories and concepts applied to most aspects of urban planning. These aspects cover land use, urban spaces, structures, facilities, city form, services, public spaces, transportation networks, and the environment. The Theory of Reasoned Action holds that emotions can influence attitudes. It addresses the positive aspects (such as happiness) and negative aspects (such as stress, fear, guilt, and complaints) that influence trust. This is a fundamental aspect of the Theory of Reasoned Action (see Table 1).

**Table 1:** The concepts used in urban planning fields.

Theory	Brief description
Theory of Planned Behavior (TPB)	Theory of Planned Behavior explains individual behavior as resulting from intentions, which are influenced by attitudes, subjective norms, and perceived behavioral control (Ajzen, 1991). The theory assumes that individuals act rationally in accordance with these three determinants of behavioral intention. Recent meta-analyses have demonstrated the theory’s continued relevance across diverse behavioral domains (Hagger et al., 2024).
Random Utility Theory (RUT)	Random Utility Theory provides a statistical framework for modeling human behavior by connecting deterministic models to probabilistic outcomes (McFadden, 1973). The randomness inherent in the utility function indicates that analysts can only examine the probability of choosing one alternative over another, rather than predicting choices with certainty (Train, 2009).
Bounded Rationality Theory (BRT)	Bounded Rationality Theory posits that human rationality is limited when making decisions (Simon, 1955). Under these cognitive constraints, rational individuals select decisions that are satisfactory rather than optimal, acknowledging the practical limitations of human decision-making processes (Gigerenzer and Selten, 2001).
Prospect Theory	Prospect Theory demonstrates that individuals evaluate losses and gains differently, making decisions based on perceived gains rather than perceived losses—a phenomenon known as “loss aversion” (Kahneman and Tversky, 1979). When presented with two identical options framed differently, individuals consistently prefer the gain-framed option. Contemporary research continues to validate this theory across various domains (Mousavi et al., 2022).
Self-Determination Theory (SDT)	Self-Determination Theory examines the motivation that drives intention formation and subsequent behavior (Deci and Ryan, 2000). The theory emphasizes that individuals are motivated by universal psychological needs to improve and enhance their capabilities. A core concept is intrinsic motivation—performing actions for the inherent satisfaction derived from the activity itself (Ryan and Deci, 2017).
Theory of Interpersonal Behavior (TIB)	The Theory of Interpersonal Behavior emphasizes the role of habit, emotion, and intention in human conduct (Triandis, 1977). It maintains that individuals possess a range of needs and expectations that govern behavior in interpersonal relations, guiding how people interact, relate, and associate with others in social contexts (Triandis, 1980).
Cognitive Dissonance Theory (CDT)	Cognitive Dissonance Theory addresses the psychological discomfort experienced when holding conflicting beliefs or attitudes (Festinger, 1957). In behavioral contexts, risk is inherently connected to concepts of threat and regret, particularly regarding negative affective experiences. Contemporary applications continue to expand the theory’s relevance (Harmon-Jones and Mills, 2019).
Collective Action Theory (CAT)	Collective Action Theory, often examined within organizational contexts and social ecological frameworks, addresses how subjective norms influence behavior (Olson, 1965). These subjective norms, analyzed through transaction cost theories, subsequently influence the formation of personal norms and collective behavior patterns (Ostrom, 1990).
Social Identity Theory (SIT)	Social Identity Theory investigates the interaction between personal and social identities in social psychology (Tajfel and Turner, 1979). The theory aims to identify and predict conditions under which individuals perceive themselves as individuals versus group members. It examines how personal and social identities affect individual perceptions and collective behavior (Turner et al., 1987).
Expected Utility Theory (EUT)	Expected Utility Theory explains decision-making under conditions of risk and uncertainty (von Neumann and Morgenstern, 1944). According to standard decision theory, when comparing alternative courses of action, individuals should choose the option with the greatest expected benefit. The principle of maximizing expected utility has broad applications (Savage, 1954).
Theory of Reasoned Action (TRA)	The Theory of Reasoned Action identifies attitude as a fundamental determinant of behavior, influenced by emotional aspects (Fishbein and Ajzen, 1975). Attitude, along with subjective norms, forms the foundation for behavioral prediction. This theory was later extended to become the Theory of Planned Behavior through the incorporation of perceived behavioral control (Ajzen and Fishbein, 1980).
Bayesian Theory	Bayesian Theory employs probabilistic analysis to understand decision-making processes (Bayes, 1763). Current research applications include conceptual models studying interactions between social power, privacy, and emotional states. The theory uses probabilistic analysis where conclusions are tied to known patterns that inform behavior (Gelman et al., 2013).
Behavioral and Cognitive Geography Theory (BCT)	Behavioral and Cognitive Geography Theory emphasizes that human–environment relationships are dynamic and bidirectional (Golledge and Stimson, 1997). Individual actions and mental states both cause and are caused by physical and social environments through ongoing, changing interactions. The theory encompasses spatial behavior and behavior in space (Kitchin and Freundschuh, 2000).
Protection Motivation Theory (PMT)	Protection Motivation Theory provides a framework for understanding responses to stimuli that individuals appraise as potential threats (Rogers, 1975). These stimuli include fear-based messages that encourage protective measures or discourage activities that may cause harm to oneself or others. The theory has been refined to include self-efficacy components (Maddux and Rogers, 1983).
Cognitive Hierarchy Theory (CHT)	Cognitive Hierarchy Theory, originating in behavioral economics, describes human thought processes in strategic games (Camerer et al., 2004). The theory includes cognitive types whose behavior ranges from random to substantively rational, with each type corresponding to the number of periods in which economic agents process new information. Empirical studies have validated the theory’s predictions (Costa-Gomes et al., 2001).
Nudge Theory (NT)	Nudge Theory focuses on how choice presentation influences decision-making outcomes (Thaler and Sunstein, 2008). The theory advocates for decision-making approaches based on people’s actual thought processes—which are often instinctive and sometimes illogical—rather than the logical and rational decision-making processes traditionally assumed. Contemporary applications include sustainability interventions (Lehner et al., 2024).
Social Ecological Model (SEM)	The Social Ecological Model considers the complex interplay between individual factors and societal relationships to understand risk and protective factors (Bronfenbrenner, 1979). The model’s nested structure demonstrates how factors at one level influence factors at other levels. Effective intervention requires working across multiple levels simultaneously (McLeroy et al., 1988).
Graph Theory (GT)	Graph Theory provides a mathematical framework used in social sciences to analyze social networks (Wasserman and Faust, 1994). It offers a pictorial representation of objects (vertices) connected by relationships (edges), enabling systematic analysis of complex social structures and interactions (Newman, 2010).
Behavioral Spillover Theory (BST)	Behavioral Spillover Theory describes how engaging in one pro-environmental behavior can promote the adoption of other such behaviors (Thøgersen and Ölander, 2003). The theory examines both positive spillover (increased probability of adopting related behaviors) and negative spillover following behavioral interventions. Research continues to explore spillover effects across contexts (Nilsson et al., 2017).
Norm Activation Theory (NAT)	Norm Activation Theory explains pro-social and pro-environmental behaviors through the activation of personal norms (Schwartz, 1977). The theory proposes that norm activation begins with an individual’s awareness of potential harmful consequences and attribution of responsibility for pro-environmental behavior. The theory has been integrated into comprehensive environmental behavior models (Stern, 2000).

The Theory of Planned Behavior (TPB) has developed beyond its original approach-top-subject criteria framework as an addition to perceived behavior control, creating a comprehensive model that accounts for individual agency and structural obstacles. Contemporary applications integrate several criteria effects, including individual value systems, individual criteria, and organizational norms from institutional contexts, shaped by comprehensive social ecological factors and cultural networks (Ajzen et al., 2024).

Bayesian models complement TPB by incorporating emotional valence and risk perception in the structure of behavior prediction. These models are responsible for comfort–discomfort reactions that affect lifestyle options and spatial behaviors, recognizing that human–environmental relations are bidirectional and dynamic through continuous spatial interaction processes (Rutter et al., 2024). Protection Motivation Theory explains danger–response mechanisms and protective behaviors, while social ecological models examine multilevel effects at the individual, mutual, and community levels. General Activation Theory addresses pro-social and environmental behavior through individual criteria activation through awareness about results and responsibility.

Communicative Planning Theory, contained in Habermas’ communication action framework, emphasizes stake engagement and collaborative decision making processes that respect all participants (Lin, 2023). Contemporary debate continues between Habermasian Communicative Planning and Maf’s agonistic planning approaches, researchers examined their relative effectiveness in various contexts.

Traditional Rational Choice models assume optimization behavior with complete information processing, while bounded rationality acknowledges cognitive limitations that constrain decision-making capabilities. Herbert Simon’s framework recognizes that individuals make satisficing rather than optimizing choices, providing a more realistic foundation for understanding human decision-making processes in planning contexts (see Table 2).

**Table 2:** Theories and urban planning models related to the behavioral fields.

Planning theories	Brief description
Communicative & Collaborative Planning Theory	Communicative and Collaborative planning (CCP) is an approach to urban planning that brings together different stakeholders and engages them in collaborative decision-making by way of respect for the stand and attitude of all concerned. It is also referred to by planning practitioners as collaborative planning or the collaborative planning model. Collaborative and collaborative planning is a prevailing theory in planning in which multiple stakeholders come together to deliberate on common concerns and apply consensus-building and public participation methods to make policy decisions. This approach seeks to balance power among participants and increase public participation. It is about why urban areas are important to social, economic, and environmental policy and how political communities can organize themselves to improve the quality of life their places (Healey, 1997). However, Healey’s (1997) analysis of the collaborative planning paradigm is extensive and synthesizes numerous themes that are strongly linked to existing issues in planning theory and practice. The issues discussed include the following: Concepts of society Power dynamics Global economic reorganization and its regional implications Environmental conservation and traditional governance arrangements and practices Organizational structure Technocratic rule and the nature of expert knowledge Mediation in conflict resolution Spatial planning The critical theory component of Friedman’s (1987) planning paradigm is framed in the extensive scholarship of social mobilization in planning practice. In this corpus of research, there exist three dominant defining features of social mobilization: Emphasizing the importance of in-place collaboration The concept of planning as an explicit form of policy Research on transformational processes
Critical Pragmatism	Critical Pragmatism (CP) is an approach to planning and public policy developed by John Forster. The basic ideas of this approach are to view planning as restructuring communication between stakeholders with divergent and conflicting interests and significant disparities in power and influence. In this approach, the planner is viewed as a practical professional who facilitates inclusive and participatory forms of collective action rather than as a rational actor and decision maker.
Bounded Rationality Theory	Bounded rationality (BR) is the theory that when individuals make choices, rationality is constrained, and that rational agents will therefore choose a satisfactory rather than an optimal choice. The limiting factors are the problem complexity to be decided on, the mental capacity of the mind, and decision-making time. Some social-science models of human action assume that human beings can be satisfactorily approximated or modeled as ‘rational’ in the rational choice theory sense or as modeled by Downs’s political agency. Bounded rationality is an extension of ‘rationality as optimization’ that imagines decision-making as a process of complete rationality to reach an optimal solution for the information available. Therefore, it is possible to state that bounded rationality dissolves the paradox between human behavior’s alleged complete rationality (used by other economic theories) and the essence of human perception.
Collaborative Planning Theory	Collaborative Planning Theory addresses the common experience of community life to disclose planning issues to be addressed. It also exists in a form of direct communication with people, having a direct influence on planning outcomes. There are fewer field observations and data analysis in interactive planning because it occurs more through interpersonal interaction, typified by a two-way learning experience.

### Urban planning theories related to the behavioral concepts

Habermas’ Communicative Action Theory provides a comprehensive framework for designing public sphere deliberation through collaborative decision-making processes enhanced by technology. Habermas’s work laid out a theory of “communicative rationality,” a form of rationality that is inherent in our language use and that “carries with its [*sic*] connotations based ultimately on the central experience of the unconstrained, unifying, consensus-bringing force of argumentative speech” (Habermas Revisited Mattila, 2018). Collaborative planning emerges as a transformative planning approach where stakeholders engage in open dialogue to understand mutual interests and contextual factors behind these interests.

One of the key concepts in Habermas’s theory is the notion of communicative rationality. Communicative rationality is the idea that communication should be governed by rational principles of argumentation and justification (PHILO-notes, 2024). Habermas established four validity claims for ideal discourse in collaborative planning: sincerity (authenticity of expression), legitimacy (appropriateness of normative context), comprehensibility (clarity of communication), and truth (accuracy of propositional content).

Critical Planning Theory, as developed by Friedmann (1987), operates within a social mobilization framework characterized by three elements: emphasis on collaborative efforts as change catalysts, recognition of planning as a political phenomenon involving power relationships and governance models, and engagement in transformational initiatives promoting structural and sociological change.

Herbert Simon introduced the term ‘bounded rationality’ as shorthand for his proposal to replace the perfect rationality assumptions of homo economicus with a concept of rationality better suited to cognitively limited agents (Stanford Encyclopedia of Philosophy, 2024). This framework addresses the gap between theoretical assumptions of perfect rationality in economic models and actual human cognitive limitations. The empirical study of human behavior from the mid-20th century to date has mainly been developed by examining the bottlenecks of the psychology of decision making (PMC, 2023). Bounded rationality recognizes that optimal decision-making is constrained by computational limitations and information processing capacity, leading to satisficing rather than optimizing behavior.

### Urban planning domains and behavioral concepts

Urban planning is a key discipline in shaping human behavior. In spite of its increasing recognition in the expanding agenda of interdisciplinary studies, a need is felt to clarify and conceptualize the interfaces of various aspects of urban planning and of human conduct. The main strength of this review is the formulation of a conceptual framework to categorize and evaluate the literature in the rapidly emerging field of study in urban planning with regard to promoting and facilitating various human behaviors. Seven distinct domains were identified in which planning can best encourage and augment human behaviors. These domains are presented in Table 3 below:

**Table 3:** Behavioral theories related to the seven urban planning domains.

Urban planning domains (*)	Theories	Behavioral theories, models, rules, and related concepts	
Built environment + Urban form + Infrastructure services + Urban landscape + Public spaces + Urban fabrics of housing forms + Urban design	Behavioral/Social Theories	01	Planned Behavior Theory
		02	Random Utility Theory
		03	Bounded Rationality Theory
		04	Probability Theory
		05	Self-determination Theory
		06	Theory of Interpersonal Behavior
		07	Cognitive Dissonance Theory
		08	Theory of Collective Action
		09	Social Identity Theory
		10	Expected Utility Theory
		11	Bounded Rationality Theory
		12	Rational Action Theory
		13	Behavioral Geography Theory
		14	Protection Motivation Theory
		15	Hierarchical Behavioral Model of Cognition
		16	Thrust Theory
		17	Social Ecological Model
		18	Standard Activation Theory
		19	Graph Theory
		20	Theory of Behavioral Spillover
	Planning theories	01	Communicative and Collaborative Planning Theory
		02	Critical Pragmatism
		03	Incrementalism and muddling through, according to the Bounded Rationality Theory
		04	Interactive Planning Model
	Urban design theories	01	Mental Image of the City – Kevin Lynch
		02	Critical Pragmatism
		03	Standards for Urban Design
		04	Theory of Modern Urban Experience
		05	Walkability Index and Walking Score
	Transport theories	01	Choose between types of transportation and transit
		02	The conventional model: the Four-Step Travel of the Urban Transport Planning System.
	Geographic theories	01	Central Place Theory
		02	Cellular Automata Model Rules of Cell Behavior
	Urban economic theories	01	Burgess Model or Concentric Area Model
		02	The Urban Economic Theory
	Utility theories	01	The Expected Utility Theory
		02	The Random Utility Theory
		03	The User Equilibrium Theory

#### Human behavior and urban form

The vision here is to re-launch behavior-centric urban studies that are human flourishing, and public space-anchored, as well as foster a move towards appreciation of places, not objects, and inter-profession practice. There also exists a multi-dimensional relationship between structure in a city and potential in public life.

It is a widely held belief in urban planning and design studies that built environment design influences the well-being and quality of life of individuals and communities. However, there are contrasting views on whether such environments have a bearing on human actions, in consideration of the fact that perhaps such environments have a bearing on achieving desirable societal outcomes. Historian John Archer argued that human conduct was determined by urbanization as well as building design (Christopher, 2002). Stepping back from determinism in relation to the environment, any presumption that the built environment determines or alters social behavior cannot help but be disconfirmed. If the physical environment makes good behavior unachievable, then behavior itself becomes unachievable, as in Alexander Christopher’s book, *The Nature of Order* (Christopher, 2002). In addition, Archer’s (2005) argument gains stronger footing when considered from the opposite perspective. Reaffirming this, it could be emphasized that comprehension and understanding of behavioral processes leads to city-making and planning that are more responsive to human needs.

Ecological contingency has been addressed throughout much written work: a concept looking at the probabilistic correlation of the physical environment and behavior. While elements of the physical environment are available to conception by everyone, layout, situation, and facility arrangements mean that there are a range of types of behavior, some of which are more likely than others. While Richard Sennett sees urbanism as a model of human comprehension of social complexity and of acquisition of empathetic understanding (Sennett, 1992), Henri Lefebvre’s interest in elements of towns refers to potentialities of action, whether through expression by individuals or by individuals in aggregate. Both, however, perceive the significance and interrelatedness of urbanization, urban form, social processes, and conduct (Lefebvre, 1992).

#### Human behavior and the built environment

Built environments consist of two components: spatial conditions and social conditions. Social conditions are those where we interact and interact with other individuals (social interaction), and spatial involvement relates to physical properties of built environments such as space, size, and object locations (or object locations) and information. There are also settings where individuals perform activities in spatial conditions of a specific content of the setting. Spatial conditions and social content generate a special fundamental relation that works together. An organized setting is the core of built environments where individuals perform a chain of interrelated routine activities that organize their daily patterns of conduct. Everyone performs these activities in organized settings in a predictable, regular fashion

#### Human behavioral factors and urban physical infrastructure services

Physical infrastructure services employed by individuals are primarily composed of flow and fixed layout configurations, but human attitudes and behaviors towards these types of services are not automatically guided by knowledge of how these configurations are set up or run. An appreciation of such a perception-system attribute divergence forms a central aspect of effective service planning, operation, and design. Interactions of human and physical infrastructure systems with electricity, transport, and water supply are widely considered a cornerstone in urban planning to project perceptions of service in urban space. Different human behaviors around utilization of infrastructure are, in turn, identified based on potential perceptions, attitudes, and preferences towards these services.

A vast corpus of literature lays the basis upon which human factors of choice in infrastructure are known. It has been specialized into service types so that much of what determines perceptions of risk is explored in infrastructure risk perception literature. Broad categories of human conduct towards these services were found to encompass comfort, convenience, accessibility, availability, environmental, social, and ethical compatibility, safety, as well as security. Social conduct towards infrastructure also manifests in appreciation of the service provided by it. Comfort and convenience manifest, in general, in avoiding congestion where possible, road/rail system choice factor, and route selection. Safety includes trip safety, i.e., exposure to natural hazards, something that forms a significant factor in transport choice. Accessibility to public transport includes railway, public transport, station, and road infrastructure, something that is needed by many individuals.

To understand what motivates individuals to use services in the way that they do, there needs to be a body of knowledge about what underlying perceptions, attitudes, and beliefs are involved with these services and why these factors link to behavior. Over time, new standards of design can be applied to enable user behavior at scale to secure infrastructure (Hurtado, 2018).

The importance of infrastructure and of “path of least resistance” to make pro-environmental actions possible cannot be overemphasized in this context and needs to find its place in action agendas of future urban development and planning by governments. The work by Kristie Nissen and colleagues focuses specifically on the environmental aspect by considering how variations in opportunities for being able to act sustainably, i.e., increasing the number of bicycle lanes in a city, shape the uptake of sustainable actions such as cycling by individuals. The authors used Copenhagen, where cycling culture has been undergoing a transformation, as a test case. The model was experimentally tested by examining what happens when a cycling versus driving culture develops in a city by simulation.

The statistics reveal how linear expansions in pro-environmental opportunities–more bike paths in Copenhagen, for example–can have much more significant impacts on take-up of sustainable practice than is commonly assumed, driven by the fact that when the environment enables take-up of a given practice by a person. This impacts not only the practice of that single individual but also that of others, as they can replicate and learn from that practice (Christiansen et al., 2017).

#### The influence of the urban landscape on social behavior

Among urban studies problems, there are some that are fundamental, such as the influence of the urban environment on humans’ intellect, conduct, and mental characteristics. Urban environment is a byproduct of the interrelation between a city-forming factor and urban society, and it describes such things as civil society, the evolution of human beings, economic conditions, tastes, aesthetics, etc. One of the most real disputes in ecology, as well as landscape theory, lies in whether landscapes influence human behavior or not, as human behavior forms through the influence of planetary environment forces. When human beings appreciate well-planned and well-put-into-space landscapes, and human beings are cooperative, landscapes are living, variegated, and strongly influence human behavior. Any change in environment, as well as taste, leads to a change in social behavior. Human nature comes from geographic nature, and its nature is formed by this; place has a secondary role to play, while urban planning has a more important one.

Motlock (2010) explained that, to produce psychologically fit urban spaces and not confused ones, we must make things obvious and evoke an emotive reaction in the observer. We must recognize that scenes exist and their meanings will be interpreted. This feeling is a widespread phenomenon and a summation of discrete elements. Considering this knowledge, we might be aware of a cognitive concept known as place, and we are able to make meaningful as well as psychologically fit spaces come true. Adibi and Azmi (2011) explained environmental perception, response, and recognition as a process by which meaning might be read from environmental space suggestions. Impact is a choice a human being makes regarding a scene. Perception is a judgment that a scene evokes through a mental depiction of a space by an observer, and meaning is put upon it.

We should appreciate the spirit, inspiration, and research behind space creation and make our judgment based upon its outcome so that we get as wide a perspective as possible of knowledge and meaning. Our ability to analyze facts and attribute meaning to facts, nonetheless, has limits. Karimi (2010) explained that humans need two things from the environment: the first is for it to be tangible and satisfying (and the environment needs protection and peace); the second is that a human being needs the environment to be a room where human beings can experience the environment and pursue their need for inquiry and analysis. If these two are attained, then human beings can learn something new and enhance their intellectual awareness within the environment.

After a shape has been formed, all combine to form a pattern. Complexity of perception then decreases, and hence, ability to visualize decreases information load. Patterns and mental images have been memorized from past experiences. All objects present in space, at the top of space, and at the edge of space, objects included; objects present in the frame are all components of the visual scene. So, to have a sense of a space is to assess if all stimuli complement each other and are making the same impression; if so, then the space has a strong, definite sense of place.

Space then turns into a cohesive, complete figure, and all the elements present in it come together. In actuality, a space never has a notion of place. If the elements of space do not come together, space then becomes discordant. It never has a sense of a common view, but appears to be turbulent, restless, and therefore creates a feeling of disorientation and does not provide a notion of place.

#### Human behavior and public spaces

Human conduct, experience, and public space social interaction are described in terms of mental activity influenced by varying features of public spaces. These features are physical, social, cultural, or sensory but have in common that they can have an influence on people’s conduct and experience in a public space. Urban designers and urban planners are professionals who are tasked with detailing, creating, and maintaining the look, feel, and aesthetic of public spaces.

Abraham Maslow, in his A Theory of Human Motivation, also remarked that behaviorism was a force both in motivation as well as in conditioning individuals to act in a specified manner. Maslow’s hierarchy of needs theory makes physiological, biological, or aesthetic needs (to be safe, to belong, and to self-actualize) determinants of human motivation. Drawing upon determinants revealed by applying Maslow’s hierarchy of needs, taking determinants’ applicability to built environments into account, it would be justifiable to believe that human actions result from exposure to physical and environmental parameters in public spaces. The physical aspects of public spaces include parameters such as built forms, streets, terrain, and people, while the environmental parameters include parameters such as light, sound, and temperature. Such a consideration of the complex correlation between human beings and surrounding environments has been presented before, coming to be known as environmental psychology (Lang, 1991).

It is the responsibility of the urban planner, urban designer, and public authority to promote human environments through creating realistic pedagogical strategies, design, policy, planning, and approach, incorporating knowledge of environmental psychology (Rasoulpour et al., 2018). There are also unique aspects of a building’s exterior that may have a potential influence on human behavior. Such exterior features may include height, opacity, architectural detail placement, and nature, such as landscaping.

Urban designers and urban builders are formally tasked with designing and constructing physical environments. However, a significant disparity exists between the two professions. Designers tend to be visual thinkers and practical problem-solvers, often focusing more on function than aesthetics. In contrast, urban planners are educated to be highly verbal and conceptually oriented (Sommer, 1984). Both designers’ goals, as well as those of urban planners, are to design settings that do not infringe on other people’s rights, while, in turn, being a reaction to the surrounding environment as well as human actions. They are therefore needed to possess a proper understanding of environmental psychological aspects. Urban planners, designers, as well as policymakers, should also benefit from, as well as apply, concepts of environmental psychology to design public settings that are visually salient, thus achieving more desirable outcomes.

#### Human behavior and urban fabrics of housing form

There is a strong relationship between the psychology of human beings and its integration with the built environment when writing about urban space. A core issue arises when there is a disconnect in communication both among people and between people and spatial design. This disharmony often occurs when city planners fail to take into consideration human beings, planning, and designing urban space without researching its influence on urban space growth. The quality of life as well as the well-being of a human being are determined by satisfaction levels in regard to housing as well as other characteristics:

- Physical scales recognizable in park provision, as well as servicing node provision, in a community.
- Determinable social characteristics based on a sense of belonging to the community.
- Determinable individual characteristics based on homeownership and years lived in a place.

Despite social variations among individuals of a single society, an identical pattern of approach to actions towards a given situation by these individuals was revealed, reflecting their culture. The extent to which a community fulfills an individual’s needs as well as desires is measured in residential satisfaction (Lu, 1999). The extent to which these needs are achieved develops from an individual’s assessment of what society offers, both in terms of materials, society, as well as individuals. They include a high-quality built environment, communal facilities, housing quality, network of interactions, sense of ownership, and one’s norms and values being accepted (Fahim et al., 2022).

Housing satisfaction matters because societal unhappiness has a potential impact upon a human being’s psychological wellbeing and quality of life (Braubach, 2007). Aside from residential choices by inhabitants to vacate the area, several factors comprising residential satisfaction have been identified in various studies, and significance being given to physical built-up areas like parks, amenities, and housing has been emphasized in various studies. Other studies (Adriaanse, 2007) have identified factors like a sense of community and social support or individual factors like length of residence and homeownership (Campbell, 2008). An in-depth examination of literature also identified a wide array of formal factors influencing residential satisfaction, a sense of community, a sense of belonging, and a sense of place. They have a clear impact on human behavior. They added that persons who have a high level of membership have enhanced social and psychological wellbeing since membership provides a sense of purpose, meaning, and significance.

Belonging has also been defined by community involvement and fear of crime, physical issues, community design, layout, residency, and marriage. When individuals belong to an area, they become more committed to a place as well as form close relationships with individuals around them (Newman and Kenworthy, 2006). They do not feel alone there; something that makes them more content as individuals is acquiring a feeling of being in a group where they belong. Belonging has a set of determining factors that outline belonging to an area as well as having a positive attitude towards a place.

#### Human behavioral dimension and urban design

Growing interest in urban design’s “human behavior dimension” reflects a strong, consolidated call for more “urban quality.” There is a clear connection between human contributions to urban space and ideas about creating lively, secure, green, and healthy urban space. Successful urban space is not merely a functional space but a pleasure space. It not only becomes a habit for users but also provides users with the right to accomplish public mastery by attending, adapting, and ascribing meaning to urban space (Simoes Albrecht, 2010). It is therefore likely that what is most significant is to reframe and adjust urban design practice to transform public life and facilitate user behavior. Rogers clarified that our urban quality of life needs to enhance urban planning and design responses of urban public space to focus on overall quality of life, environment, health, and safety for all.

A review of environmental psychology revealed that the field of behavioral psychology deals with relations and interactions between individuals and peripheral environmental physical components and is interested in how human behavior and emotions are influenced by the environment from a theoretical point of view. City design and urban planning, as a point of confrontation and a close network of relations between citizens, can have significant potential to guide urban habits. The human being, being the most impactful factor in urban social life, has access to the highest level of influence from the environment. Human conduct in environmental psychology is shaped by a variety of factors such as environmental physical components, symbolic information, design information, and an environmental atmosphere.

Human action, experience, and interaction with public spaces are understood to stem from mental processes influenced by specific qualities of space. These qualities (sensory, cultural, or social) share a common feature they have the potential to shape people’s behaviors and experiences in the public space By rethinking the ‘human behavioral dimension’ of urban public spaces, we see they are a reflection of the city’s excitement, tension, and vitality, acting as a unifier, not a divider, of its spatial, but also its social, dimensions—the street is a public space rightfully owed to everyone. People are personally connected to the public spaces they live in and where they interact with each other daily. Urban ideologies reveal that interest in the form of public spaces was driven by a passion to improve the quality of life.

Urban space never fails to leave room for the generation of phenomena of conduct that are in themselves a byproduct of the aspect of identity and interrelation based on human and societal activity. It is therefore necessary in urban planning and design that human activity is considered in terms of how it acts and responds to the ambiance and environment of a space.

Seen in Table 4, Lynch, in his Mental City Image, defines and describes five elements that constitute the mental concept of a city: nodes, paths, neighborhoods, landmarks, and edges. The concept of Eyes on the Street, explained by Ewing & Clemente, defines activity in the city streets that makes the flow of the street safe and secure. It argues that if streets are filled by people, our streets are safer to travel through because if a single individual is threatened, then the eyes of the street are poised to rescue and save them from harm. Modern Urban Experience Theory outlines the chances of concrete sensory modes to sense and to document urban environments. In contrast, concepts of various indicators and measures have been set to measure walking conditions, stimulating modern cities and encouraging them to develop favorable walking conditions in a bid to enable human physical activity, enhance human happiness, dissuade traffic, and promote a healthy urban environment. This paves the way for an argument that urban design must become more sensitive to all of the favorable spatial, experiential, and social places and environments used by people who have chances to stimulate favorable interaction.

**Table 4:** Urban design theories and models related to the behavioral field.

Urban design theory	Brief description
Five elements of a city	Lynch’s (1960) cognitive mapping theory identifies five fundamental elements that structure urban mental images: paths (channels of movement), edges (boundaries), districts (recognizable areas), nodes (focal points), and landmarks (reference points). These elements collectively form the “imageability” of urban environments and influence wayfinding behavior.
Five measures of urban design	Ewing and Clemente (2013) provide standardized measurement protocols for five key qualities of urban design: imageability (memorability and distinctiveness), visual enclosure (spatial definition), human scale (comfortable proportions), transparency (visual connection between interior and exterior spaces), and complexity (visual richness). These measures enable objective assessment of urban design quality.
Theory of modern urban experience	Simmel’s (1903) sociological theory examines how urban environments shape psychological adaptation and social behavior. The theory describes how city dwellers develop protective mechanisms against overstimulation while maintaining capacity for meaningful urban engagement. It emphasizes the interplay between individual perception and collective urban experience.
Eyes on the street theory	Jacobs (1961) theory of natural surveillance argues that street safety emerges from continuous informal observation by residents and users. The concept emphasizes that mixed-use, high-density neighborhoods with diverse activities create optimal conditions for “natural owners” to monitor public spaces, thereby enhancing security through passive surveillance rather than formal control mechanisms.
Walkability index and walk score	Complementary measurement systems for pedestrian-friendly environments. The Walkability Index (WI) evaluates physical environmental features such as sidewalk connectivity, street design, and land use mix (Frank et al., 2010). Walk Score (WS) provides accessibility-based measures focusing on proximity to amenities and services (Walk Score Professional, 2011). Both metrics support evidence-based urban planning for sustainable transportation and public health outcomes.

However, enormous volumes of behavioral data from mobile devices as well as from social media provide insight that would otherwise be inaccessible through data from surveys, small-scale studies, field work, and focus groups. Social media data provide spatial-temporally correlated digital traces of human urban space utilization from direct access to virtual aspects of urban living, such as people’s habits as well as preferences based on these data. Urban planning researchers can analyze human activities and disclose key urban planner concepts from these data. Urban planners, as well as researchers in Geospatial Data Science Lab (GDSL), are using place data power also to facilitate inquiry into human behavioral patterns using a longitudinal settlement mobility dataset to address the issue of planning as well as routing future cities to model check-in activity (mobility) among different loci (points of interest).

They also created a geospatial data science structure that transformed check-in points and raw user destination streams into illustrations of the changing forms and characteristics of city neighborhoods. Through analysis of vast data using geographic data science methods, scholars were able to analyze the form structure of the city as well as its activity space involvement and its contexts of conduct. Table 4 also shows an outline of urban design along with model factors pertaining to the field of conduct.

### The role of biophilia in human-centered urban planning

Biophilia, a term popularized by biologist Wilson (1984), refers to congenital human affinity for natural environment and living systems. This concept suggests that humans have evolved into close contact with nature and therefore are entitled to a deep-seated psychological need to interact with green vegetation and a life-filled environment.

The principle of biophilia has since become a fundamental idea in environmental psychology, which indicates a design philosophy that wants to once again achieve an environment created with human evolutionary preferences in mind (Kellert, 2008). In terms of urban planning, biophilic design provides a practical framework to understand how natural elements contribute to mental health, cognitive restoration, pro-environmental behavior, and social harmony (Browning et al., 2014).

Many empirical studies have shown that in urban settings, vegetation, water features, daylight, and organic forms reduce access to stress, improve attention, and increase emotional welfare (Frumkin et al., 2017; Lopes et al., 2025; Lencastre et al., 2023). These results are especially true for dense urban environments where sensitive overload and environmental scarcity are common. Many planning models have begun integrating biophilic principles at the neighborhood and city levels, including strategies such as green corridors, urban forests, roofing gardens, and bio-revised architecture (Beatley, 2011).

These initiatives not only serve ecological stability goals but also act as behavioral interventions. In this way, biophilia pulls environmental aesthetics and psychological functionality in the human-focused plan.

By including biophilia in the current structure, environmental behavior strengthens the experiential and restorative dimensions of urbanism. This confirms the argument that cities should not only be efficient or functional but also emotionally resonant and psychologically helpful. As urban design moves rapidly towards focusing on overall well-being, biophilic strategies provide a tangible route to recreate urban form, improve spatial equity, and increase flexibility in both personal and community behavior.

## Discussion

A systematic structure has been established to examine interrelations between urban planning and behavioral concepts in seven planning areas: urban form, built environment, urban physical infrastructure services, urban landscapes, public places, urban environment, and urban design. This structure integrates social behavior principles, planning principles, and urban design principles to understand how various behavioral determinants affect urban planning strategies. The urban form significantly shapes the resident personality through urban environment and continuous interaction between humans. Contemporary urban design theory and planning exercises believe that the environment created by environmental design affects community life and good to a great extent. The interaction between urbanization, urban form, social mobility, and behavioral factors creates an environment that supports specific behavioral tendencies, including fueling the feeling of location, social interaction, and especially sociological settings within communal attachment (Chen et al., 2023).

Environmental psychology provides an ideological structure to understand why human behavior should be integrated into environmental design. Knowledge gaps about human behavior, approaches, and values present important challenges to architects and urban planners who design the created environment. Taking advantage of behavioral science knowledge enables urban planners and designers to merge social and personal aspects of human experience (Liu and Zhang, 2024).

The physical environment affects humans while simultaneously being converted to meet human needs and conduct. Spacious references, combined with social materials, establish specific relations within organized environmental settings where individuals engage in regular activities that define their daily conduct. The spatial pattern of behavior needs to be considered alongside individual and user space behavior requirements.

**Figure 2:**
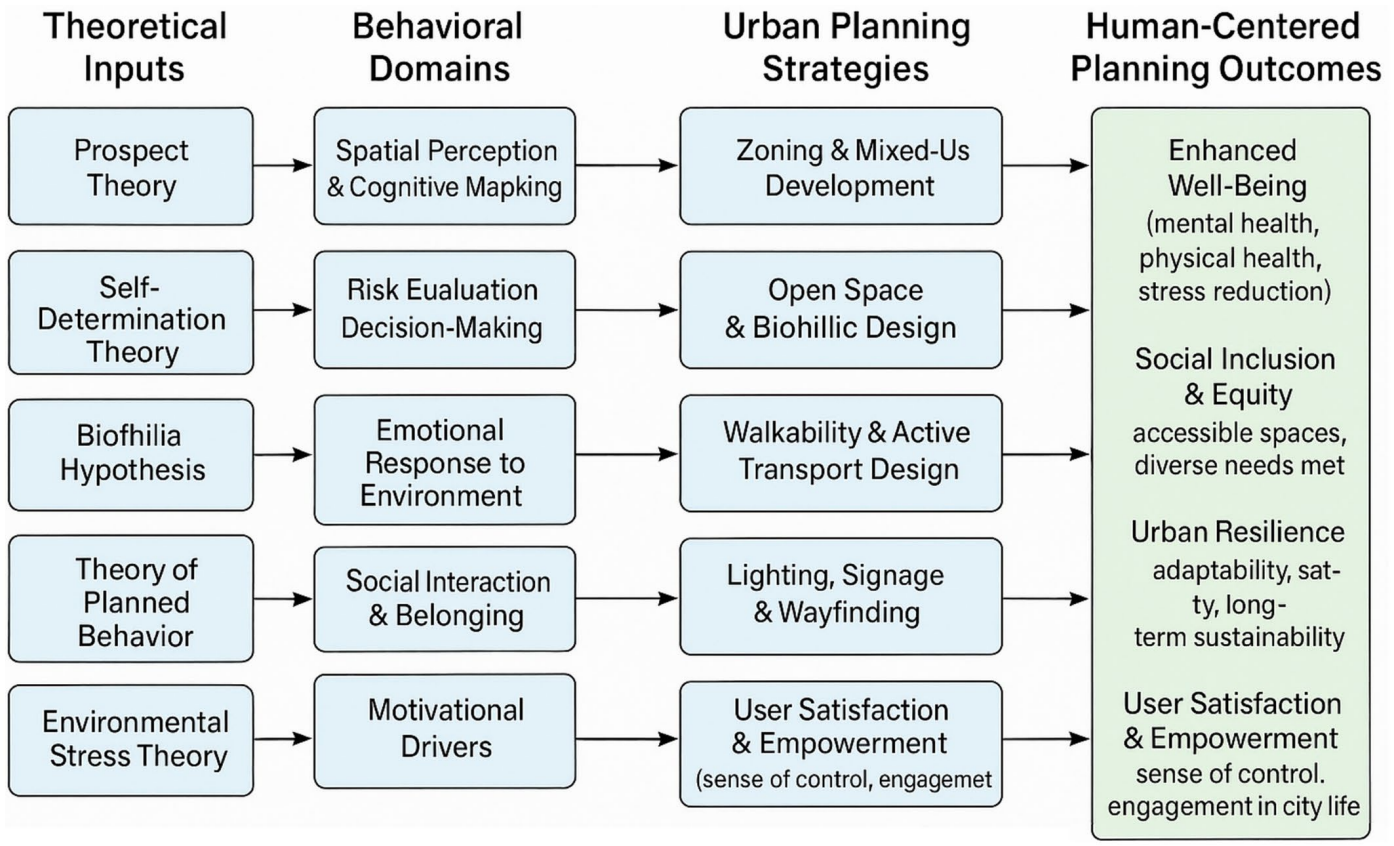
Conceptual framework for integrating behavioral science into human-centered urban planning.

The early urban form model, which includes the concentrated field models and sector models of Hoyat and Burges, includes behavior and its relationship with the urban environment, group succession ecological approach, and assumption of housing market investors, although they show a lack of clear behavioral details. Behavioral science enables understanding of the current social patterns and a more accurate forecast of future consequences of urban design and planning proposals. Environmental psychology reduces the uncertainty of urban designers and planners, which reduces uncertainty in determining the needs of the user by improving designs through the interpretation of environmental study findings. The urban environment affects human beings’ mental, cognitive, and behavioral aspects through a city’s factor generation through negotiations of urban society (see Figure 2).

## Conclusion

The purpose of this study was to understand the complementary relationship between human behavioral principles and urban planning by examining and analyzing the behavioral principles applied in urban planning-condemned subjects. Various planning studies and principles were considered that address a variety of human functions in urban environments. Through comprehensive analysis, the study shows that there is a tendency to analyze a variety of functions in areas related to urban planning with significant breadth, which can be explained through various theoretical outlines, as mentioned in later sections.

### Key findings

- *Literature integration and future directions*: A careful examination of literature on urban planning and fundamental concepts highlights the importance of current and future thinking about urban planning responsibilities and the need to guide urban planning towards dependence on planning principles based on the behavior complex.
- *Theoretical Outline Application Theory*: Different approaches are provided to understand the relationship between behaviors at all levels of society (from personal behavior to social systems). Their basic components serve as a suitable frame of reference to study and guide the urban planning process, and in urban planning suggested the possible applications of this “behavior approach.”
- *The psychological impact of urban planning*: The way cities are planned or designed is directly related to the psychology of each person in their special context. Poorly employed cities can obstruct economic activity and increase social disturbance and moral decline, while well-employed cities are economically rich, socially and culturally harmonious, and environmentally supported, durable societies.
- *Behavioral manifestation in urban environments*: This provides a platform for the expression of persistent urban environment behavioral patterns that themselves are products of identification factors and interactions because of human and social activities. Therefore, in urban planning and design processes, it is necessary to consider human behavior and how individuals experience and function within their surroundings and environment.
- *The predictive capabilities of behavioral science*: Behavioral science allows us to understand the modern patterns of social life and the environment in which we reside, thus expanding our ability to predict the future effects of urban design. The knowledge gained through environmental psychology enables urban designers and planners to reduce ambiguity in their design options, which respond to user needs during the planning and design stages. Additionally, incorporating findings from human behavior reviews can contribute to design reforms by integrating these insights into design proposals and planning recommendations.
- *Professional responsibilities and social change*: The contribution of fundamental issues and intellectual challenges or behavioral approaches is clear in the role that urban planners and planning processes themselves should play in the direction of social change and the impact it will have on human behavior through urban planning processes. This approach will significantly add to the nature of the profession and its full understanding of its responsibilities.
- *Urban planning as a behavioral tool*: Urban planning and design are key tools for shaping places where people gather and interact socially; they can play an effective role in directing human behavioral patterns.

This study demonstrates that integrating behavioral theory with urban planning practices offers a comprehensive framework for understanding and influencing human behavior in urban environments, ultimately contributing to more effective and responsive urban design and planning strategies.

### Future directions and research implications

This study contributes to behavioral urban planning by demonstrating how cognitive biases and environmental psychology shape urban behaviors while establishing a framework for incorporating these insights into practice.

Emerging technologies are creating new opportunities for behavioral urban research. AI and machine learning enable real-time analysis of urban behavior through mobile data, IoT sensors, and social media interactions. Digital twin technologies allow us to model behavioral responses to interventions before implementation, while smart city initiatives generate datasets on mobility patterns and social interactions. These advances enable personalized urban experiences where cities adapt services to individual behavioral preferences while maintaining collective functionality.

Future methodological directions should prioritize longitudinal designs to understand how interventions evolve over time. Randomized controlled trials in urban settings can establish causal relationships between environmental modifications and behavioral outcomes. Participatory research laboratories engaging residents as co-researchers bridge academic insights with lived experiences. Virtual and augmented reality technologies offer controlled testing environments for proposed urban designs. Mixed methods approaches combining quantitative behavioral data with qualitative ethnographic insights will provide comprehensive understanding of behavior change mechanisms.

Behavioral urban research raises significant policy and ethical implications. Behavioral nudging in public spaces requires careful attention to consent, autonomy, and democratic participation. Proactive equity assessments must ensure interventions benefit all residents rather than privileging certain groups.

The outline of the decision-making should guide the planners, thus expanding our ability to predict the future impacts of urban design and planning more precisely than we are currently able to do, aligning them with effectiveness, morality, and community preferences. Integration of behavioral science requires interdisciplinary cooperation between planners, psychologists, and technologists, which requires institutional changes in educational training and professional practice. The regulatory structure must be developed to address privacy concerns, data governance, and algorithm accountability in urban environments.

## Recommendations

1. Urban planners need to systematically incorporate interdisciplinary insights from behavioral sciences—particularly environmental psychology and social geography—into planning frameworks to enhance the human responsiveness of urban environments.
2. Planning authorities should institutionalize participatory design methodologies, wherein residents are actively engaged in decision-making processes to ensure that spatial configurations align with the behavioral norms, cultural dynamics, and lived experiences of local communities.
3. Urban development strategies must prioritize pedestrian-oriented design by enhancing walkability, connectivity, and multimodal accessibility, thereby fostering pro-social behaviors, public health, and spontaneous social interactions.
4. The integration and equitable distribution of green and open public spaces should be a central pillar in urban planning policies, given their empirically proven benefits on emotional regulation, psychological well-being, and community cohesion.
5. Policy frameworks should adopt a context-sensitive approach that accounts for socio-cultural variability and localized behavioral patterns, ensuring that urban environments are socially inclusive and behaviorally adaptive.
6. It is imperative that planning interventions be accompanied by rigorous post-implementation behavioral assessments to evaluate their effectiveness, inform policy revisions, and support the development of data-driven, human-centered urban policies.

## Data Availability

The original contributions presented in the study are included in the article/supplementary material, further inquiries can be directed to the corresponding authors.
